# Graph-enhanced multimodal fusion of vascular biomarkers and deep features for diabetic retinopathy detection

**DOI:** 10.3389/frai.2025.1731633

**Published:** 2026-02-02

**Authors:** K. V. Deepsahith, Basineni Shashank, Bangipavan Kumar, Sherly Alphonse, Brindha Subburaj, Girish Subramanian

**Affiliations:** 1 School of Computer Science and Engineering, Vellore Institute of Technology, Chennai, India; 2 School of Business Administration, Penn State Harrisburg, Middletown, PA, United States

**Keywords:** contrast limited adaptive histogram equalization (CLAHE), Convolutional Neural Networks (CNNs), deep learning, MobileNetV3, retinal images

## Abstract

Diabetic retinopathy (DR) detection can be performed through both deep retinal representations and vascular biomarkers. This proposed work suggests a multimodal framework that combines deep features with vascular descriptors in transformer fusion architecture. Fundus images are preprocessed using CLAHE, Canny edge detection, Top-hat transformation, and U-Net vessel segmentation. Then, the images are passed through a convolutional block attention module (CBAM)-fused enhanced MobileNetV3 backbone for deep spatial feature extraction. In parallel, the segmented vasculature is skeletonized to create a vascular graph, and the descriptors are computed using fractal dimension analysis (FDA), artery-to-vein ratio (AVR), and gray level co-occurrence matrix (GLCM) texture. A graph neural network (GNN) then generates a global topology-aware embedding using all that information. The different modalities are integrated using a transformer-based cross-modal fusion, where the feature vectors from MobileNet and GNN-based vascular embeddings interact using multi-head cross-attention. The fused representation is then given to a Softmax classifier for DR prediction. The model demonstrates superior performance compared to traditional deep learning baselines, achieving an accuracy of 93.8%, a precision of 92.1%, a recall of 92.8%, and an AUC-ROC of 0.96 for the DR prediction in the Messidor-2 dataset. The proposed approach also achieves above 98% accuracy for Eyepacs and APTOS 2019 datasets for DR detection. The findings demonstrate that the proposed system provides a reliable framework compared with the existing state-of-the-art methods.

## 1 Introduction

Diabetic Retinopathy (DR) is a microvascular complication of diabetes and affects the retinal vasculature. This also alters the retinal characteristics, like microaneurysms and hemorrhages, which are important biomarkers for early-stage detection. Therefore, quantifiable retinal features and vascular patterns are observable in fundus images. Artificial intelligence (AI) and deep learning approaches have significantly enhanced automated screening and diagnosis, and also led to accurate DR detection (Aljohani and Aburasain, 2024). The retinal characteristics also work as an effective biomarker for systemic pathologies like hypertension, diabetes, and cardiovascular disease(CVD) (Poplin et al., 2018; Ikram et al., 2006). Based on the retinal vascular alterations, it is possible to forecast these diseases and enable interventions on time. This resulted in incorporating AI and deep learning algorithms for machine-based analysis that improved the efficacy and accuracy of retinal image-based diagnosis (French et al., 2022). Yet, it is time-consuming, subject to inter-observer variation, and not feasible in large-scale screening.

Despite the improvement in medical technology, traditional diagnostic methods are still mainly invasive, costly, and unavailable to some countries (Chang et al., 2020). Sophisticated medical facilities and professional expertise are needed for established methods such as coronary angiography, echocardiography, and cardiac MRI, thus restricting their applicability. Consequently, researchers have been driven to create new alternatives that exploit low-cost and noninvasive strategies for detecting early disease (Rim et al., 2021).

Deep learning allows for automatic feature extraction and classification, lowering the reliance on manual interpretation. Convolutional Neural Networks (CNNs) have proved enormously successful in detecting and classifying abnormalities on medical images, from tumor detection in radiology to retinal pathology detection in ophthalmology (Kermany et al., 2018).

In this proposed work, to improve classification accuracy, various image preprocessing methods, such as contrast limited adaptive histogram equalization (CLAHE), Canny edge detection, Top-hat transformation, and U-Net for vessel segmentation, are employed. A vascular graph is created from the segmented and then skeletonized images. Then gray-level co-occurrence matrix (GLCM) is used for regional feature extraction. GLCM offers texture-based features that assist in distinguishing normal and abnormal retinal patterns. Further, the fractal dimension analysis (FDA) is integrated to measure vascular complexity and structural abnormalities for the early detection of DR. Artery-to-vein ratio (AVR) is also an important biomarker to indicate DR severity. A graph neural network(GNN) embeds the vascular graph with other feature descriptors like GLCM, FDA, and AVR to create the graph-embedded features.

The segmented images are also given as input to a lightweight CNN model, MobileNetV3, which is optimized for high efficiency and low computational overhead, as the basis for an efficient and scalable automated DR detection. In contrast to traditional CNN models that require heavy computational resources, MobileNetV3 uses depth-wise separable convolutions, which greatly minimize the number of parameters without compromising accuracy. MobileNetV3 is highly suitable for real-time applications like mobile health systems and telemedicine platforms. Components like squeeze-and-excitation(SE), block attention mechanism, and dilated convolutions are also added to enhance it further in the proposed work. The SE attention mechanism recalibrates the feature maps dynamically, and hence the model focuses on critical vascular areas (Tseng et al., 2023). The dilated convolutions enhance the receptive field, which helps the model to detect the fine-grained vascular patterns, which are highly useful for identifying early disease. The transformer-based cross-modal fusion used in the proposed system helps in the fusion of deep features from MobileNetV3 and graph-embedded features. The contributions of the proposed work are listed as follows:

- **Preprocessing techniques:** CLAHE, Canny, and Top-hat transformation help in enhanced visibility of vessels.
- **Vessel segmentation:** U-Net helps in vessel segmentation, thereby boosting overall accuracy while extracting global and local features.
- **Local features extraction:** GLCM, FDA, and AVR calibration help in extracting local features.
- **Global features extraction:** MobileNetV3 and SE block attention mechanism enhance feature selection by dynamically recalibrating channel-wise feature responses, ensuring the model focuses on critical vascular regions such as microaneurysms, vessel narrowing, and tortuosity, which are the key indicators of DR.
- **Dilated convolutions:** Increases receptive field without elevating computational expense, allowing for the identification of fine retinal vascular abnormalities, including subtle vessel deformity and capillary dropout, that are frequently linked to DR prediction.
- **Convolutional block attention(CBAM) Module:** Helps in enhancing vessel structures, suppressing noise.
- **Graph-based embedding:** GNN helps in graph-enhanced feature embedding and also preserves the information about the vascular junctions and branches.
- **Cross-modal fusion:** The deep features from MobileNetV3 and graph-embedded features are fused using a transformer-based cross-modal fusion technique.

In the existing literature, several studies exist that focus on graph-based learning, multimodal fusion, and attention mechanisms for DR detection. But most of the existing models use only feature-level fusion across CNN streams and not physiological structures. Also, the graph-based approaches mostly rely on handcrafted descriptors, without vascular biomarkers. Most of the existing works treat the modalities as independent channels, without any standardized method for cross-modal interactions.

The proposed framework helps in addressing these gaps. (i) This work proposes a vascular biomarker graph in which nodes encode the descriptors, and edges model the anatomical relationships. This representation helps in capturing the disease-relevant dependencies that are not seen in other conventional attention-based fusion models. (ii) A graph-enhanced multimodal fusion module is proposed that uses a relation-aware fusion mechanism. Thus, the model learns complementary interactions between learned deep features and structured biomarker information, which is better than the existing hybrid pipelines. The proposed system also uses vascular biomarkers FDA, and the arteriolar-to-venular ratio (AVR) that captures the earlier microvascular changes due to DR. The transformer-based cross-modal fusion module has better interaction modeling that improves the robustness.

Section 2 discusses other existing works in the literature. Section 3 outlines the methodology, wherein preprocessing improves vascular structures before feeding them into a MobileNetV3-based model with dilated convolutions and SE attention. It also explains the proposed integrated methodology used for DR detection. Section 4 reports experimental results on the different datasets (Herrerot, 2022), providing metrics such as accuracy, precision, recall, and AUC-ROC scores. Section 5 concludes the findings.

## 2 Related works

(Gulshan et al., 2016) constructed a deep learning model for diagnosing DR based on retinal fundus images. The system had high specificity and sensitivity, demonstrating the viability of CNNs in automating diagnosis. The work of Gulshan et al. emphasizes the benefits of non-invasive imaging methods for high-volume screening. Limitations lie in the need for large annotated datasets and computation for training and deployment. Solutions to these might make it more viable in low-resource settings.

(Das and Pumrin 2024) investigated the application of MobileNet in the classification of retinal images to diagnose DR. MobileNet's thin model supports low-cost computation, which is useful for low-resource environments. Data preprocessing methods, such as resizing and augmentation, were demonstrated in the study to significantly enhance model performance. The study, however, did not conduct an exhaustive examination of the effects of varying preprocessing approaches on prediction accuracy, leaving it for future studies to further improve these techniques.

(He et al. 2015) presented the ResNet architecture that overcomes the problem of vanishing gradients in deep networks using residual connections. (Litjens et al. 2017) used CNN-based architectures as a building block for processing challenging medical images such as retinal scans. (Zhang et al. 2019) suggested the use of attention mechanisms with deep learning frameworks. The application of attention mechanisms improves the diagnostic performance and interpretability. (Liu et al. 2024) proposed an adversarial learning-based framework for the segmentation that leads to better feature representation and edge detection. The model was good for noisy and complicated datasets. (Huang et al. 2023) explored the contrastive learning methods to classify retinal images. It lowers the dependency on expert-annotated examples. (Aljohani and Aburasain 2024) suggested a hybrid glaucoma detection system with Random Forest and CNNs (ResNet50, VGG-16) for glaucoma detection. (Ting et al. 2017) considered the effects of automated deep learning models on early disease identification, workflow performance, and diagnostic accuracy.

Shipra et al. (2024) used explainable AI (XAI) in medical imaging. The work used Grad-CAM and SHAP values to visualize outputs that also helped the clinicians to understand and believe AI-derived predictions. The incorporation of XAI into CNN models enhanced confidence in automated diagnostic systems. Nonetheless, there were issues raised in terms of balancing explainability and predictive performance, as a few interpretable models had a slightly lower accuracy compared to their black-box variants. Future research should investigate how to improve interpretability without losing classification accuracy, perhaps through hybrid AI-human decision-making systems.

(Ronneberger et al. 2015) proposed the U-Net architecture, which has been well used in medical image segmentation, including retinal vessel extraction. The experiment proved that skip connections and upsampling policies of U-Net were better at maintaining spatial details than standard CNNs, leading to better segmentation accuracy. The model's capability of performing well on small datasets was especially useful in medical applications. Nevertheless, the experiment showed a reliance on the quality of the datasets and domain-specific fine-tuning. Table 1 gives a detailed review of some of the existing works.

**Table 1:** Summary of the DR Detection methods in literature.

**No**.	**References**	**Focus**	**Dataset**	**Source**	**Methodology**	**Findings**	**Keywords**
1	Pratt et al., 2016	DR Classification	EyePACS	Variable quality	CNN + data augmentation	Robust	Retinal images, classification, CNN
2	Gulshan et al., 2016	DR Detection	EyePACS, Messidor-2	High-resolution fundus images	Inception-V3 CNN	High sensitivity	DR, deep learning, screening
3	Voets et al., 2019	Cross-domain DR performance	EyePACS, Messidor	Mixed clinical	Comparative CNN analysis	Performance drop in domain shift	Domain adaptation
4	Lam et al., 2018	DR Lesion Detection	Messidor	Good quality images	Transfer learning (ResNet)	Enhanced microaneurysm detection	Transfer learning
5	Li et al., 2019	DR Grading	DDR Dataset	Clinical dataset	Attention-based CNN	Attention maps	DR grading
6	Fang and Qiao, 2022	Early DR Detection	DIARETDB1	Medium-quality	Hybrid DL + handcrafted features	Improved early lesion detection	Hybrid ML
7	Dixit and Jha, 2025	DR Staging	APTOS, Messidor	High-quality image	EfficientNet classifier	Lightweight model	EfficientNet
8	Keel et al., 2019	DR Screening	Primary dataset	Low and variable real-world images	DL-based clinical screening system	Real-world clinical workflows	Screening system

The transformer architectures have recently enhanced multimodal learning approaches. (Shamshad et al. 2023) in their survey have highlighted the ability to model the cross-modal interactions better than CNNs. (Zhou et al. 2023) introduced a transformer-based model that processes radiographs, text, and laboratory data using intra- and inter-modal attention, which performed better than image-only pipelines. (Warner et al. 2024) examined multimodal machine learning in clinical biomedicine, indicating the fusion and alignment problems that actually motivate for graph-aware and transformer-based models. (Dong et al. 2025) proposed a multimodal transformer system that combines fundus images with clinical data for DR diagnosis, showing the importance of cross-attention compared to retinal and systemic features to improve the performance.

Haq et al. (2024) reviewed the DR detection models, indicating the vision transformers' good performance. (Bhoopalan et al. 2025) proposed a task-optimized vision transformer (TOViT) for DR detection. (Mutawa et al. 2024) designed a CNN-based DR staging model with CLAHE and discrete wavelet transform to pre-process the images. (Senapati et al. 2024) reviewed CNNs, hybrid, and transformer-based methods, which support the use of transformer-based multimodal fusion for DR detection. The deep learning-based models have displayed potential in classifying retinal disease (Gulshan et al., 2016; Kermany et al., 2018), they tend to be based on large annotated images and are less interpretable (Wang et al., 2020; Litjens et al., 2017). General models such as EfficientNet and ResNet need domain-level fine-tuning (He et al., 2015), while attention-based algorithms are computationally expensive. To overcome such limitations, the proposed method promotes contrast with CLAHE (Zuiderveld, 1994) and obtains clinically meaningful biomarkers, such as AVR (Seidelmann et al., 2016), FDA, and GLCM-based texture features (Haralick et al., 1973), while maintaining physiological relevance.

## 3 Proposed methodology

**Figure 1:**
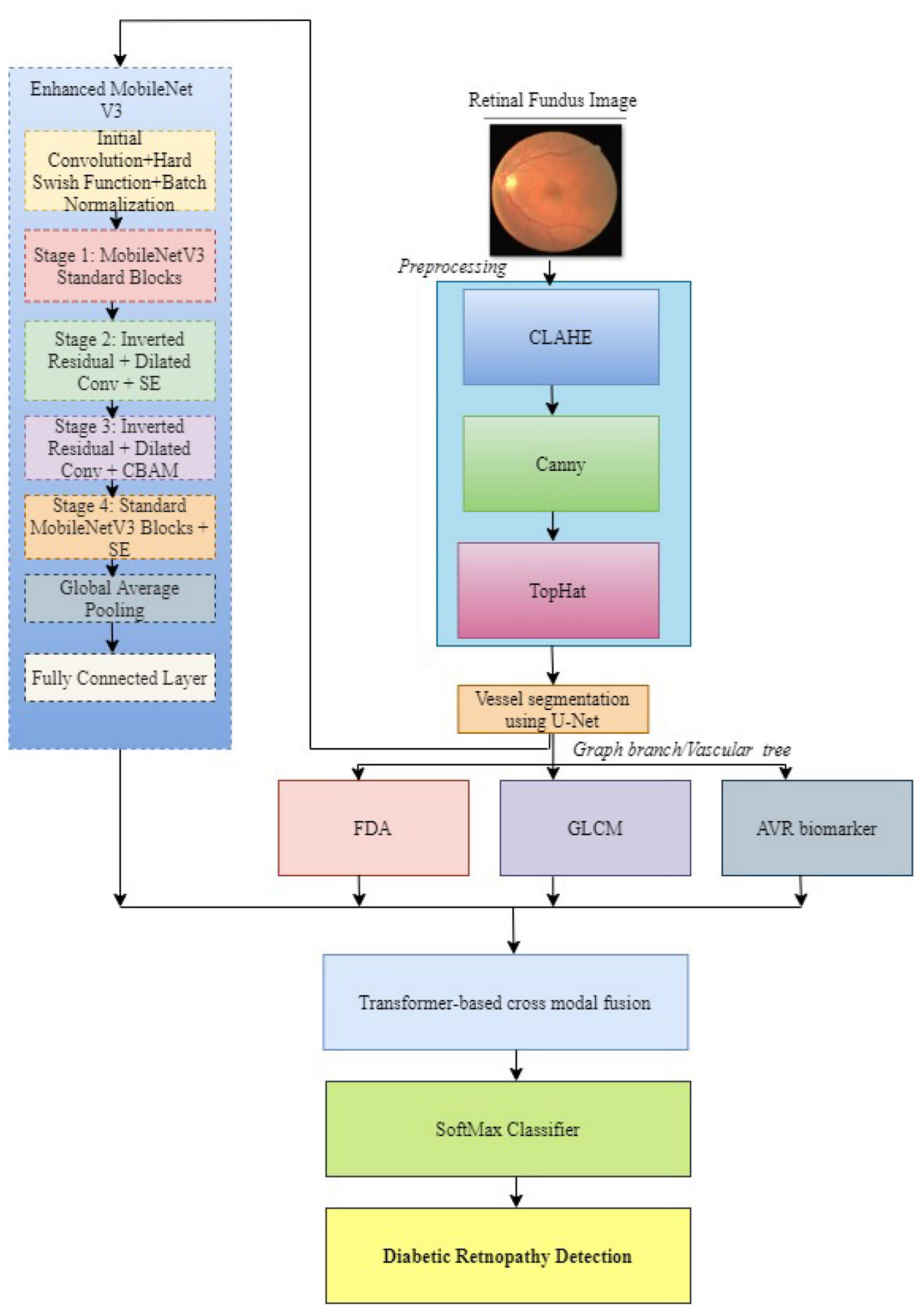
Overview of the proposed multi-modal framework with preprocessing pipeline, deep-features, graph-embedded features, and transformer-based cross-modal fusion.

The goal of the proposed system is to enable an integrated system for the efficient and interpretable diagnosis of DR detection by analyzing retinal fundus images. The system, illustrated in Figure 1, is designed around deep learning and traditional image processing techniques to capture both macro-level and micro-level features in retinal vasculature. Initially, the system acquires high-resolution retinal images, which are subjected to a series of preprocessing steps aimed at enhancing visual quality and suppressing noise. The use of CLAHE and Canny edge detection helps in improving the vessel contrast and delineation.

After preprocessing, the U-Net creates a segmented image that's passed into an enhanced MobileNetV3 network. To complement the learned representations, handcrafted features are extracted from the same preprocessed and segmented images. These include GLCM descriptors that capture vascular texture properties and FDA, which quantifies the complexity of vessel branching. The AVR is also computed, and a vascular embedding is created using GNN. Then this is fused with deep features from MobileNetV3, using transformer-based cross-modal fusion that enhances the model's interpretability and robustness.

### 3.1 Dataset

Each of the DR tests in the Messidor-2 dataset consists of two macula-centered eye fundus images, one for each eye. The dataset only contained photos that were macula-centered. There are 874 examinations (1748 pictures) in Messidor-2. The excess black background has been removed from this preprocessed version of the Messidor-2 dataset, which is accessible at Messidor-2. The MESSIDOR-2 DR grades are the source of the DR grades (https://www.kaggle.com/datasets/mariaherrerot/messidor2preprocess).

Blindness detection was separated into groups for training, validation, and testing in the APTOS 2019 dataset. The Asia Pacific Tele-Ophthalmology Society 2019 Blindness Detection (APTOS 2019 BD) collection contains 3662 samples collected from numerous individuals in rural India. The Aravind Eye Hospital in India organized the dataset. The fundus images were collected from a number of locations and conditions over a long period of time. The samples were then analyzed and categorized by a group of trained medical experts using the International Clinical DR Disease Severity Scale (ICDRSS) as a reference. According to the scale system, the APTOS 2019 BD samples are divided into five groups: proliferative DR, mild DR, moderate DR, severe DR, and no DR (https://www.kaggle.com/datasets/mariaherrerot/aptos2019).

The International Clinical Diabetic Retinopathy (ICDR) grading scale, which divides retinal fundus pictures into five DR severity categories, is used in the EyePACS dataset. A healthy retina with no discernible microaneurysms or lesions is represented by class 0 (No DR). Only microaneurysms, which manifest as tiny red spots on the retina, are seen in Class 1 (Mild DR). Microaneurysms are included in Class 2 (moderate DR), which also includes moderate vascular anomalies or other hemorrhages. Intra-retinal microvascular abnormalities (IRMA) and multiple hemorrhages are characteristics of class 3 (severe DR); however, proliferative DR is not present. The most advanced stage, known as Class 4 (Proliferative DR), is characterized by neovascularization and vitreous or preretinal hemorrhages, increasing the risk of visual loss (https://www.kaggle.com/competitions/diabetic-retinopathy-detection). In the experiments, a five-fold cross-validation is used.

To further support vessel segmentation and feature validation, an additional publicly available dataset, the retina blood vessel dataset (Wagih, 2023), is incorporated. These datasets provide a broader spectrum of retinal characteristics and enhance model generalization (https://www.kaggle.com/datasets/abdallahwagih/retina-blood-vessel).

### 3.2 Preprocessing techniques

Preprocessing improves image quality and emphasizes diagnostically relevant structures. This study employs a series of transformations to highlight blood vessels, reduce image noise, and extract spatial texture information.

#### 3.2.1 Vessel visibility enhancement using CLAHE

**Figure 2:**
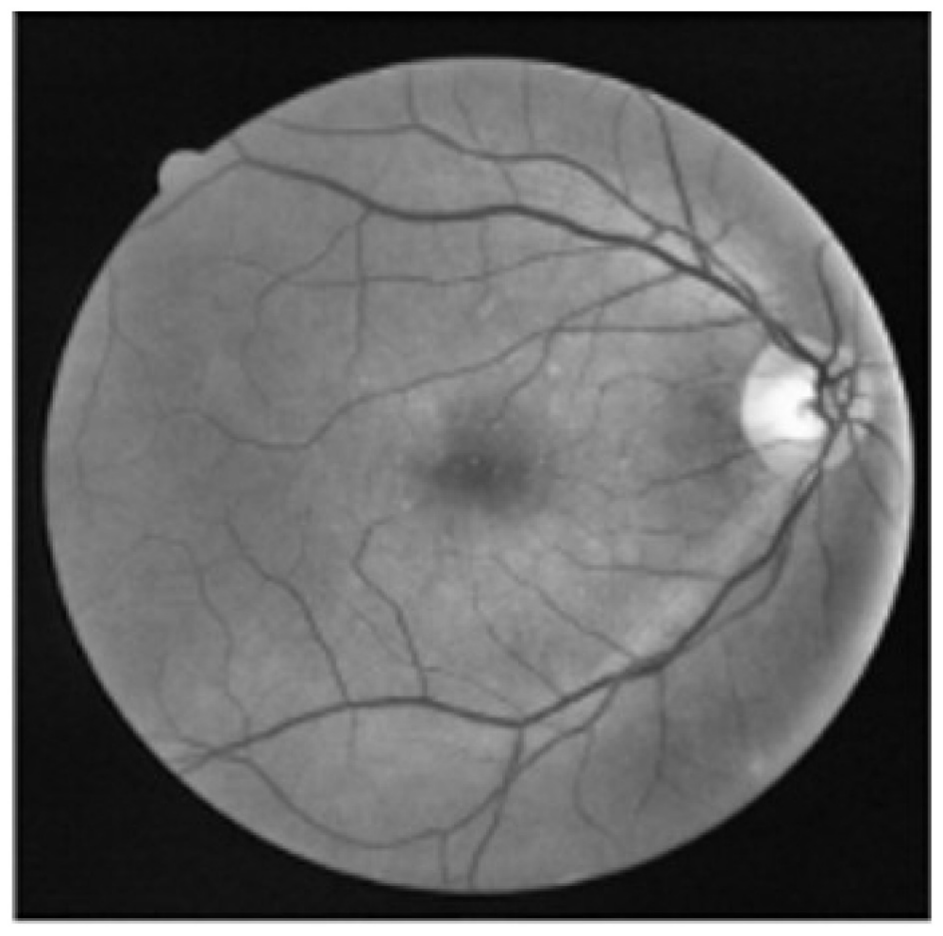
Illustration of vessel visibility enhancement in retinal fundus images using CLAHE.

CLAHE improves local contrast by equalizing intensity values in small image tiles, avoiding over-enhancement and preserving fine details as in Figure 2. The transformation is computed using:


$$\begin{array}{l} T\left(x\right)=\frac{CDF\left(x\right)-CDF_{\min}}{M-CDF_{\min}}\times \left(L-1\right) \end{array}$$ (1)

where:

- *CDF*(*x*) is the cumulative histogram value at intensity *x*,
- *CDF*_min_ is the minimum histogram value in the tile,
- *M* is the number of pixels per tile,
- *L* is the maximum pixel intensity.

#### 3.2.2 Highlighting vessel boundaries using Canny algorithm

**Figure 3:**
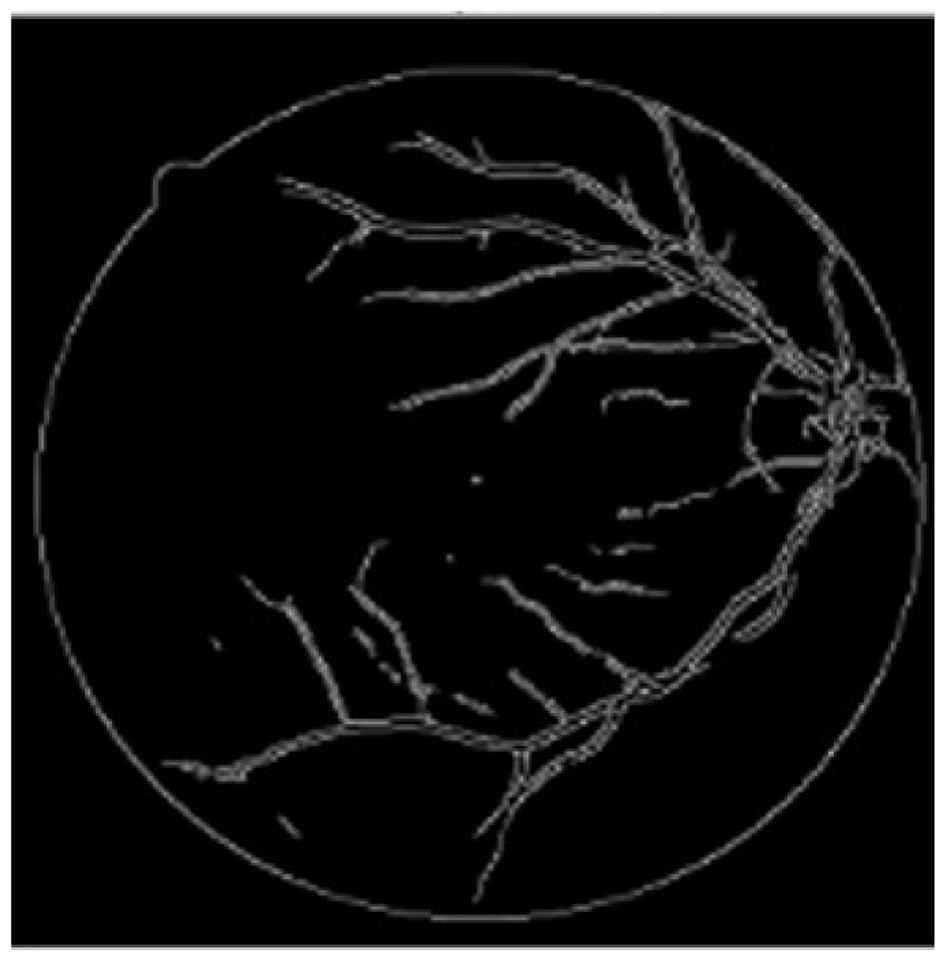
Result of Canny edge detection and highlighting vessel boundaries in retinal fundus images.

The Canny algorithm identifies edges by detecting gradients and applying non-maximum suppression. The steps include Gaussian smoothing and gradient estimation as in Figure 3:


$$\begin{array}{l} G\left(x,y\right)=\frac{1}{2\pi \sigma^{2}}e^{-\frac{x^{2}+y^{2}}{2\sigma^{2}}} \end{array}$$ (2)

$$\begin{array}{l} G=\sqrt{G_{x}^{2}+G_{y}^{2}} \end{array}$$ (3)

where *G*_*x*_ and *G*_*y*_ are the gradients in the *x*− and *y*− directions.

#### 3.2.3 Morphological vessel enhancement (Top-hat transform)

The Top-hat transform isolates small, bright objects such as vessels. The mathematical formula is given as:


$$\begin{array}{l} T_{\text{top-hat}}\left(I\right)=I-\left(I{}^{\circ}B\right) \end{array}$$ (4)

where ° denotes morphological opening.

#### 3.2.4 U-Net for segmentation

The combination of preprocessing techniques leads to:

- Enhanced visibility of fine vascular patterns.
- Suppression of imaging artifacts and irrelevant background.
- Improved feature extraction by the deep learning backbone.

These effects collectively improve the system's diagnostic accuracy, specificity, and generalizability for real-world screening applications in DR detection. Here, the U-Net is used for segmentation purposes before extracting the features using MobileNet and handcrafted features. It also removes the noisy background and improves accuracy and interpretability (Ronneberger et al., 2015). The segmented vasculature is then converted into a skeleton representing the branching topology. Here, the nodes represent the anatomical points, the edges represent the vessel segments, and the attributes are the features. The graph representation helps to preserve the local and global properties.

### 3.3 Feature extraction blocks

The feature extraction blocks used help in the extraction of both the semantic features and fine-grained statistical cues from retinal images. After preprocessing, the segmented image from the U-Net is given to the MobileNetV3, which helps in capturing vessel tortuosity, branching, and lesion features. An SE attention block with the dilated convolution layer improves the focus on relevant areas within the image. Simultaneously, the segmented image is skeletonized into a vascular graph, and the features are extracted using GLCM and FDA. GLCM extracts the second-order texture information, such as contrast, correlation, and homogeneity, and FDA computes the complexity and self-similarity of the vascular structures. AVR, which is a vital biomarker used in the proposed approach, is also computed. The features are embedded in a graph-based representation using GNN, along with the topology information about the junctions and branches. The deep feature and the graph-embedded features are then fused using a transform-based cross-modal fusion, which is then passed to a classification head that performs the final prediction.

This proposed approach has both the strength of deep features and handcrafted features that improve the sensitivity even to subtle vascular variations.

### 3.4 Enhanced MobileNetV3

The enhanced MobileNetV3 extracts the deep features regarding microaneurysms, hemorrhages, exudates, and vessel abnormalities, which are the primary indicators of DR.

#### 3.4.1 Dilated convolution

**Figure 4:**
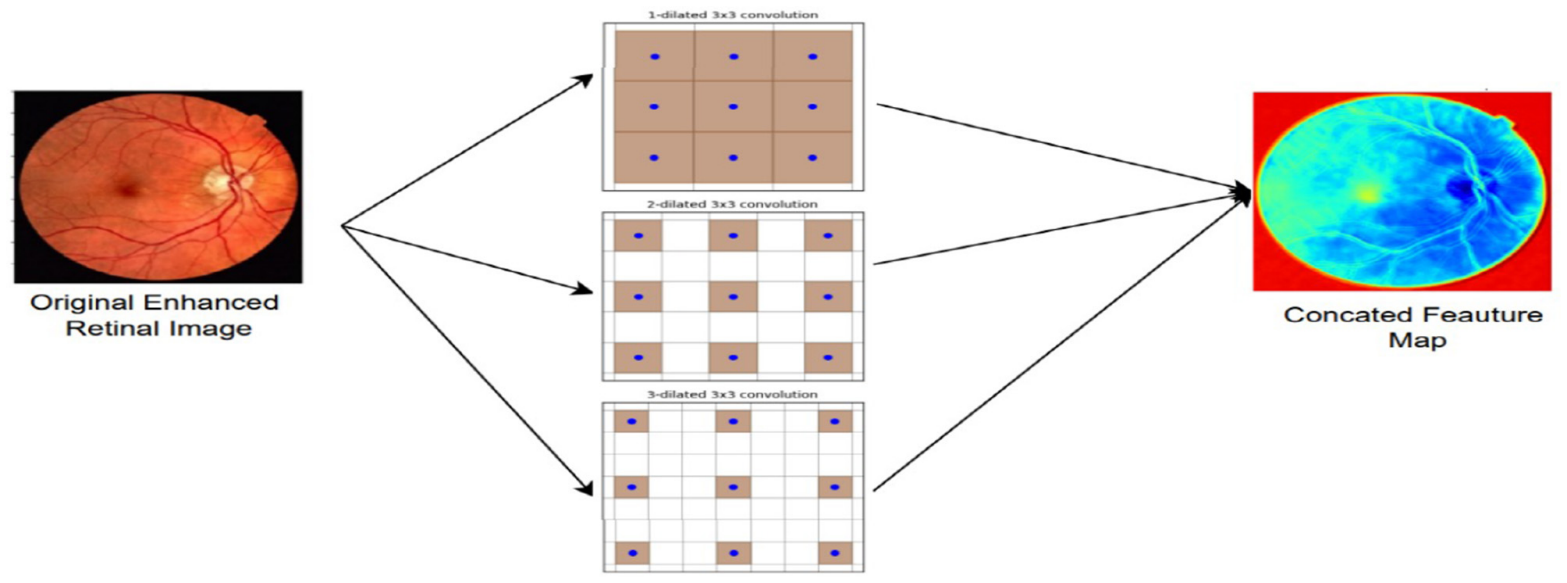
Dilated convolution used in MobileNetV3.

Pooling is typically performed after a primary convolution operation to reduce dimensionality and strengthen the local features. Pooling also enhances the receptive field, and more global features can be extracted. However, the fine-grained information is lost in the feature maps, which can reduce image recognition accuracy. Without pooling, the receptive field may still be too limited, as it would prevent the extraction of larger spatial relations. With pooling being included, the receptive field of the convolutional kernel is larger, allowing for broader feature extraction. To overcome the disadvantage of pooling, dilated convolution was introduced as shown in Figure 4. This technique modifies the convolution process by introducing gaps (or dilation) among kernel elements, increasing the receptive field without losing the resolution of the feature maps. Unlike pooling, dilated convolution doesn't alter the sizes of input and output feature maps; therefore, no spatial information is lost.

Dilated convolution has several advantages. One, through the addition of a dilation rate, the receptive field is widened without sacrificing resolution, with the relative spatial relation between pixels remaining intact. Two, through the addition of more dilated convolutions with varied rates, multiscale contextual features are obtained. Three, computational cost is relieved because the receptive field is widened without new parameters added (Yu and Koltun, 2015). Algebraically, dilated convolution is written as:


$$\begin{array}{l} z\left(p,q\right)=\sum f\left(p+d\cdot h,q+d\cdot j\right)\cdot g\left(h,j\right) \end{array}$$ (5)

where:

- *p, q* are the horizontal and vertical coordinates in the feature map.
- *h, j* are the coordinates in the convolution kernel.
- *f* represents the feature map values.
- *g* represents the convolution kernel values.
- *d* is the dilation rate, determining the spacing between kernel elements.

#### 3.4.2 Squeeze-and-Excitation block (SE) attention mechanism

**Figure 5:**
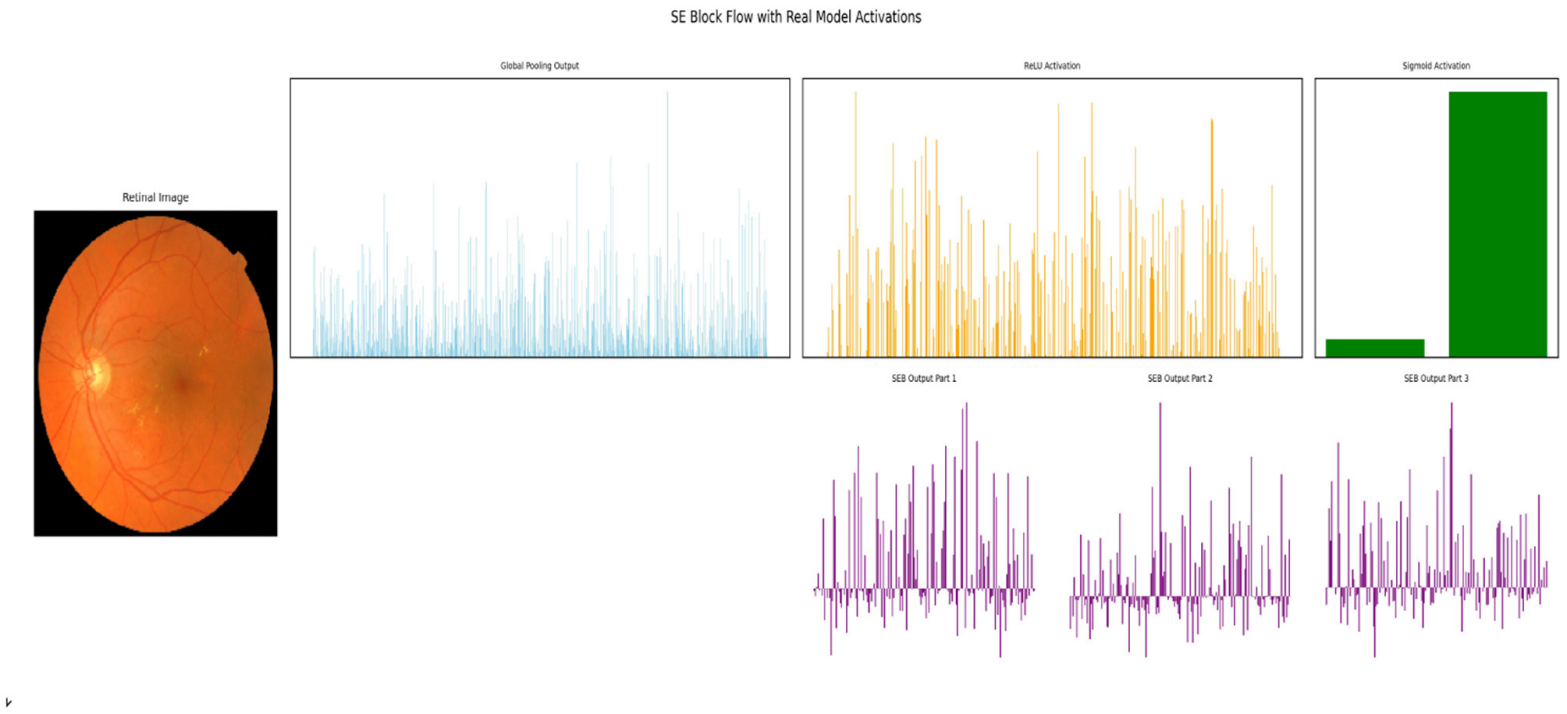
Squeeze-and-Excitation Block used in MobileNetV3.

The SE block improves the performance of MobileNetV3 by adaptively recalibrating channel-wise feature responses. SE blocks, as in Figure 5, enhance the informative ones while suppressing less relevant channels. This helps in detecting the vascular abnormalities in retinal images, like vessel narrowing, tortuosity, and microaneurysms, better.

The squeeze and excitation step compresses the spatial features (of size *H*×*W*) in each channel using Global Average Pooling (GAP) (Jin et al., 2022), resulting in single descriptor per channel:


$$\begin{array}{l} z_{c}=\frac{1}{H\times W}\overset{H}{\underset{i=1}{\sum }}\overset{W}{\underset{j=1}{\sum }}X_{c}\left(i,j\right) \end{array}$$ (6)

Here, *X*_*c*_(*i, j*) is the activation at pixel (*i, j*) in channel *c*.

The channel-wise descriptors are given to a bottleneck consisting of two fully connected (FC) layers with non-linear activations (ReLU and sigmoid) that result in a learned attention weight for each channel:


$$\begin{array}{l} s=\sigma \left(W_{2}\cdot \delta \left(W_{1}\cdot z\right)\right) \end{array}$$ (7)

Where:

- *W*_1_ and *W*_2_ are the weight matrices,
- δ is the ReLU activation function,
- σ is the sigmoid activation function where output is in the range [0, 1].

The original feature maps are then scaled by the learned weights.


$$\begin{array}{l} {\hat{X}}_{c}=s_{c}\cdot X_{c} \end{array}$$ (8)

where *s*_*c*_ is the learned attention weight, and *X*_*c*_ is the original feature map.

The dilated convolutions capture the multiscale spatial context without affecting the resolution. The global average pooling used in SE blocks aggregates global channel-wise statistics. This ensures that high-level contextual cues are significantly enhanced without compromising spatial details. This highlights the diagnostic features and suppresses the noisy channels.

The MobileNetV3 used in this framework has dilated convolutions and SE-block attention mechanisms. The use of SE blocks enhances feature selection. The non-linear(NL) functions, such as Hard-Swish (HS) and ReLU activation functions, enhance the efficiency. CBAM helps in focusing better on relevant features.

### 3.5 Vascular graph construction

The U-Net model segments the vessel structures, and the binary vessel map was obtained using morphological thinning. Bifurcation points and crossovers are identified using connectivity analysis. Each location, based on proper retinal vasculature, is a node, and the edges represent vessel continuity. Artery-vein (A/V) classification is identified using discriminative descriptors, local intensity statistics, and vessel width. GLCM-based texture descriptors are computed along each segment, and a lightweight classifier assists this identification. Each node is encoded with FDA, AVR, and GLCM-derived texture measures. Vessel segments belonging to the same branch are assigned a consistent A/V label, which is later used to augment the node attributes and other features. The graph was then processed using a GNN to create a global embedding summarizing morphology, topology, and descriptors.

The preprocessed retinal fundus image is defined as


$$\begin{array}{l} I:\text{Ω}\subset {\mathbb{R}}^{2}\to \mathbb{R}, \end{array}$$ (9)

where Ω is the retinal image domain. After vessel segmentation, the vessel set is obtained as:


$$\begin{array}{l} S=\left\{x\in \text{Ω}:\text{vessel}\left(x\right)=1\right\}. \end{array}$$ (10)

The operator Skel(·) produces a reduced skeleton structure using:


$$\begin{array}{l} K=Skel\left(S\right)\subset \text{Ω}, \end{array}$$ (11)

which preserves the vascular topology.

For each skeleton pixel p∈K, 8-connected neighborhood is defined as


$$\begin{array}{l} N\left(p\right)=\left\{q\in K:||p-q{||}_{\infty }=1\right\}. \end{array}$$ (12)

Nodes *V* are indicated as


$$\begin{array}{l} \;V=\{p\in K:|N(p)|=1\text{ (endpoints) or}\\ |N(p)|\geq 3\text{ (junctions)}\}. \end{array}$$ (13)

The edges *E* are the maximal simple paths in K between two nodes, and all intermediate pixels have a degree |N(p)|=2.

The retinal vasculature is represented as a graph as:


$$\begin{array}{l} G=\left(V,E\right), \end{array}$$ (14)

where the nodes are the endpoints/junctions, and edges are the vessel segments.

Let *A* ∈ {0, 1}^|*V*| × |*V*|^ represent the adjacency matrix with


$$A_{ij}=\{\begin{array}{ll} 1 & \text{if }(i,j)\in E,\\ 0 & \text{otherwise}, \end{array}$$ (15)

and *D* = diag(*d*_*i*_) be the degree matrix with $d_{i}=\underset{j}{\sum }A_{ij}$. The normalized adjacency is defined as:


$$\begin{array}{l} Ã=A+I,\;\tilde{D}=\text{diag}\left(\underset{j}{\sum }{Ã}_{ij}\right), \end{array}$$ (16)

This is used in graph neural network (GNN) processing (Kipf and Welling, 2017).

Also, deep retinal features are extracted using a MobileNet backbone enhanced with CBAM. To integrate the information from deep features and vascular-graph embeddings, a transformer-based cross-modal fusion was used. The MobileNet-CBAM feature vector and the GNN-derived vascular embedding are different modalities, and multi-head cross-attention helps in modeling the interactions. The final representation has both structural vascular biomarkers and appearance-based cues. This final fused feature vector is given to a Softmax classification layer to predict DR severity.

### 3.6 GLCM and FDA

Gray-Level Co-occurrence Matrix (GLCM) texture descriptors are calculated as:


$$\begin{array}{l} \text{Contrast}=\underset{i,j}{\sum }{\left(i-j\right)}^{2}G\left(i,j\right), \end{array}$$ (17)

$$\begin{array}{l} \text{Correlation}=\frac{\sum_{i,j}\left(i-\mu_{i}\right)\left(j-\mu_{j}\right)G\left(i,j\right)}{\sigma_{i}\sigma_{j}}, \end{array}$$ (18)

$$\begin{array}{l} \text{Energy}=\underset{i,j}{\sum }G{\left(i,j\right)}^{2}, \end{array}$$ (19)

$$\begin{array}{l} \text{Homogeneity}=\underset{i,j}{\sum }\frac{G\left(i,j\right)}{1+|i-j|}, \end{array}$$ (20)

where *G*(*i, j*) is the normalized GLCM, and μ_*i*_, σ_*i*_ are the mean and standard deviation of row *i*. The RGB histogram is calculated as:


$$\begin{array}{l} H_{c}\left(i\right)=\underset{x,y}{\sum }\delta \left(I_{c}\left(x,y\right)-i\right),\;c\in \left\{R,G,B\right\} \end{array}$$ (21)

where δ is the Kronecker delta, and *I*_*c*_(*x, y*) is the pixel intensity at (*x, y*) for channel *c*. GLCM captures the texture patterns regarding microaneurysms and hemorrhages effectively. Here, FDA is a non-invasive biomarker capturing retinal abnormalities. The box-counting technique is used to compute the fractal dimension in FDA as:


$$\begin{array}{l} FD=\underset{\epsilon \to 0}{\lim}\frac{\log N\left(\epsilon \right)}{\log\left(1/\epsilon \right)}, \end{array}$$ (22)

where *N*(ϵ) is the number of boxes of size ϵ used to cover the vessel's structure, regaining the vascular branching's complexity.

### 3.7 AVR calibration

The vessel caliber is computed using the arteries and veins identified in the zone of 0.5–1.0 optical disc diameters from the disc margin. The central retinal arteriolar equivalent (CRAE) and central retinal venular equivalent (CRVE) are estimated using the Parr-Hubbard formulas:


$$\begin{array}{l} CRAE=\sqrt{\left(0.87\cdot D_{1}^{2}+1.01\cdot D_{2}^{2}\right)}, \end{array}$$ (23)

$$\begin{array}{l} CRVE=\sqrt{\left(0.72\cdot d_{1}^{2}+0.91\cdot d_{2}^{2}\right)}, \end{array}$$ (24)

where *D*_1_, *D*_2_ are the highest arteriolar diameters, and *d*_1_, *d*_2_ are the highest venular diameters. A lower AVR reflects narrower arterioles associated with significantly increased risk (Ikram et al., 2006; French et al., 2022). The formula for computing the AVR is given as:


$$\begin{array}{l} AVR=\frac{CRAE}{CRVE}. \end{array}$$ (25)

Canny edge detection enhances the accuracy of delineating vessel boundaries, especially in low-contrast or noisy fundus images, by finding the edges accurately. Hence, a more reliable segmentation of arterioles and venules is possible, which results in accurate CRAE/CRVE calculation (Seidelmann et al., 2016; McGeechan et al., 2008).

The AVR is a crucial retinal biomarker for DR detection. Dilated convolution modules in the architecture expand the receptive field without losing spatial resolution, thereby capturing multiscale vessel structures like fine capillaries and larger branches needed for robust vessel segmentation and AVR estimation. The inclusion of Frangi filters helps identify broken or small arterioles. DR results in lower AVR values, due to venular widening (Islam et al., 2009; Ashraf et al., 2021). The arteriolar narrowing is seen in regions of retinal non-perfusion and increased DR severity. Wider retinal venules predict the progression of DR over time (Liu et al., 2022). AVR is a helpful quantitative indicator of microvascular alterations in DR. Fused with other features, it is more effective (Quellec et al., 2017).

### 3.8 Graph neural network (GNN) encoder

The vascular graph G=(V,E) is embedded with regional features, such as GLCM descriptors, and global vascular biomarkers such as AVR, and FDA features (Kipf and Welling, 2017).

Let $X\in {\mathbb{R}}^{|V|\times d_{x}}$ denote the node feature matrix, where each node feature vector *x*_*i*_ includes:


$$\begin{array}{l} x_{i}=[c_{i},g_{i}^{\text{con}},g_{i}^{\text{ent}},t_{i}] \end{array}$$ (26)

with *c*_*i*_ the vessel caliber, $g_{i}^{\text{con}},g_{i}^{\text{ent}}$ GLCM contrast/entropy, and *t*_*i*_ the artery/vein label.

Each GNN layer embeds the node information across vessel connections:


$$\begin{array}{l} H^{\left(0\right)}=X,\;H^{\left(\ell +1\right)}=\sigma \left({\tilde{D}}^{-\frac{1}{2}}Ã{\tilde{D}}^{-\frac{1}{2}}H^{\left(\ell \right)}W^{\left(\ell \right)}\right), \end{array}$$ (27)

where Ã is the adjacency with self-loops, $\tilde{D}$ the degree matrix, *W*^(ℓ)^ trainable parameters, and σ(·) a non-linearity.

Along with the local encoding, this model also incorporates the global vascular biomarkers:


$$\begin{array}{l} s=[\text{AVR},D_{\text{FD}},{ḡ}^{\text{con}},{ḡ}^{\text{ent}}], \end{array}$$ (28)

where AVR is the arteriovenous ratio in the optic disc annulus, *D*_FD_ the fractal dimension of the vascular tree, and ḡ^con^, ḡ^ent^ are mean GLCM descriptors computed over the vasculature.

After *L* GNN layers, the node embeddings ${\left\{h_{i}^{\left(L\right)}\right\}}_{i\in V}$ are pooled to form a graph-level representation:


$$z_{G}=\rho (\left[h_{i}^{\left(L\right)}:i\in V\right])∥s,$$

where ρ(·) is an attention pooling, and ∥ denotes aggregation with the handcrafted global biomarker vector *s*.

Thus, the final embedding $z_{G}$ has both the vascular structural information acquired using GNN and clinically interpretable global biomarkers (AVR, FDA, and GLCM).

### 3.9 Transformer-based cross-modal fusion

To aggregate all the descriptors, a cross-modal fusion is done using a transformer encoder (Lu et al., 2019).

- $z_{\text{img}}\in {\mathbb{R}}^{d_{\text{img}}}$: fundus image embedding from MobileNet.
- $z_{G}\in {\mathbb{R}}^{d_{G}}$: vascular graph embedding from the GNN encoder.
- $s\in {\mathbb{R}}^{d_{s}}$: handcrafted vascular descriptors (AVR, fractal dimension, GLCM features).

All features are projected into a manifold of dimension *d*:


$$\begin{array}{l} t_{\text{img}}=P_{\text{img}}z_{\text{img}},\;t_{G}=P_{G}z_{G},\;t_{\text{stat}}=P_{\text{stat}}s, \end{array}$$ (29)

where $P_{\text{img}},P_{G},andP_{\text{stat}}$ are the trainable projection matrices.

The input token sequence is constructed as


$$\begin{array}{l} T_{0}=[t_{\text{cls}};t_{\text{img}};t_{G};t_{\text{stat}}]\in {\mathbb{R}}^{4\times d}, \end{array}$$ (30)

where *t*_cls_ is a learnable classification token.

Each transformer block uses multi-head self-attention (MHSA) succeeded by feed-forward layers:


$$\begin{array}{l} \text{MHSA}\left(T\right)={\text{Concat}}_{h=1}^{H}(\text{softmax}\left(\frac{Q_{h}K_{h}^{⊤}}{\sqrt{d_{h}}}\right)V_{h})W^{O}, \end{array}$$ (31)

where $Q_{h}=TW_{h}^{Q}$, $K_{h}=TW_{h}^{K}$, $V_{h}=TW_{h}^{V}$.

After *B* transformer layers, the fused representation is obtained from the classification token:


$$\begin{array}{l} t_{\text{fused}}=t_{\text{cls}}^{\left(B\right)}. \end{array}$$ (32)

### 3.10 Prediction

DR is predicted using the classifier as in :


$$\begin{array}{l} ŷ=\text{softmax}\left(W_{c}t_{\text{fused}}+b_{c}\right). \end{array}$$ (33)

This architecture enables joint reasoning across image-level features, vascular topology, and handcrafted descriptors, improving robustness and interpretability.

## 4 Experimental results and analysis

The datasets used in the experiments, such as APTOS 2019, EyePACS, and Messidor-2, have different grading protocols, image quality, and image acquisition techniques. Class-balanced augmentation is used to manage the imbalancing problem. Messidor-2, APTOS2019, and EyePACS use different DR grading schemes, and therefore, the labels were standardized to a unified 5-class International Clinical Diabetic Retinopathy (ICDR) scale to ensure consistency. To handle dataset heterogeneity, preprocessing and normalization techniques are applied to three datasets. The preprocessing pipeline, which includes CLAHE, Canny edge detection, and Top-Hat filtering, is applied to all datasets. The parameter values can be adjusted to handle the variations in illumination, resolution, and image quality across datasets. Here, all experiments employ 5-fold cross-validation for all experiments, with patient-level splitting applied for Messidor-2 and EyePACS. All images from a single patient stay in the same folder, which prevents cross-patient data leakage. For APTOS2019, stratified 5-fold image-level splitting is used while maintaining class balance.

### 4.1 Dataset preprocessing and augmentation

To guarantee high-quality input data, CLAHE was utilized for contrast improvement to highlight fine retinal blood vessel pathology. Canny edge detection was used for accurate vessel segmentation, and morphological Top-hat filtering was employed to improve the measured vessel morphology. GLCM texture features were also used to determine spatial relationships among retinal microstructures. The FDA was utilized to estimate vascular complexity to provide a more quantitative measure of structural pathology.

Table 2 shows a comparison of three retinal image datasets. Messidor-2, being the main dataset used in this work, had the accuracy (93.8%), followed by EyePACS (98.2%) and APTOS 2019 (99.2%).

**Table 2:** Accuracy obtained by the proposed model on different datasets.

**Dataset**	**Classes**	**Images**	**Accuracy**
Messidor-2	5	1,748	93.8%
EyePACS	5	88,700	98.2%
APTOS 2019	5	3,662	99.2%

### 4.2 Model training and optimization

Training was carried out with categorical cross-entropy loss and Adam optimizer with the initial learning rate of 0.0001, which was reduced step-by-step using the ReduceLROnPlateau scheduler to avoid overfitting. Early stopping criterion tracked validation loss and stopped the training process if performance was satisfactory, avoiding repeated computation for the best convergence.

To test every component's contribution, several test runs were undertaken with and without notable enhancements like SE attention, dilated convolutions, and upgraded preprocessing. How each such component added performance is illuminated by the ablation studies (described in Section 4.5).

### 4.3 Performance metrics and evaluation

To empirically assess the performance of the aforementioned model, a set of evaluation performance measures was used, such as accuracy, precision, recall, specificity, F1-score, and AUC-ROC. Accuracy is one of the main measures of whether the model classifies DR classes correctly, but classification evaluation cannot be described at all by it, and a set of these measures is more crucial to obtaining the balance of false positives as well as false negatives. The hyperparameters used are given in Table 3.

**Table 3:** Hyperparameters used in the proposed model.

**Parameter**	**Setting**
Batch size	32
Epochs	100
Optimizer	Adam
Initial learning rate	1 × 10^−4^
Weight decay	1 × 10^−5^
Hidden dimension of transformer	256
Attention heads	4
Adam β_1_, β_2_	0.9, 0.999
Hardware	NVIDIA RTX 3090 GPU (24 GB), 64 GB RAM
Framework	PyTorch 2.1.0 with CUDA 12.2

**Figure 6:**
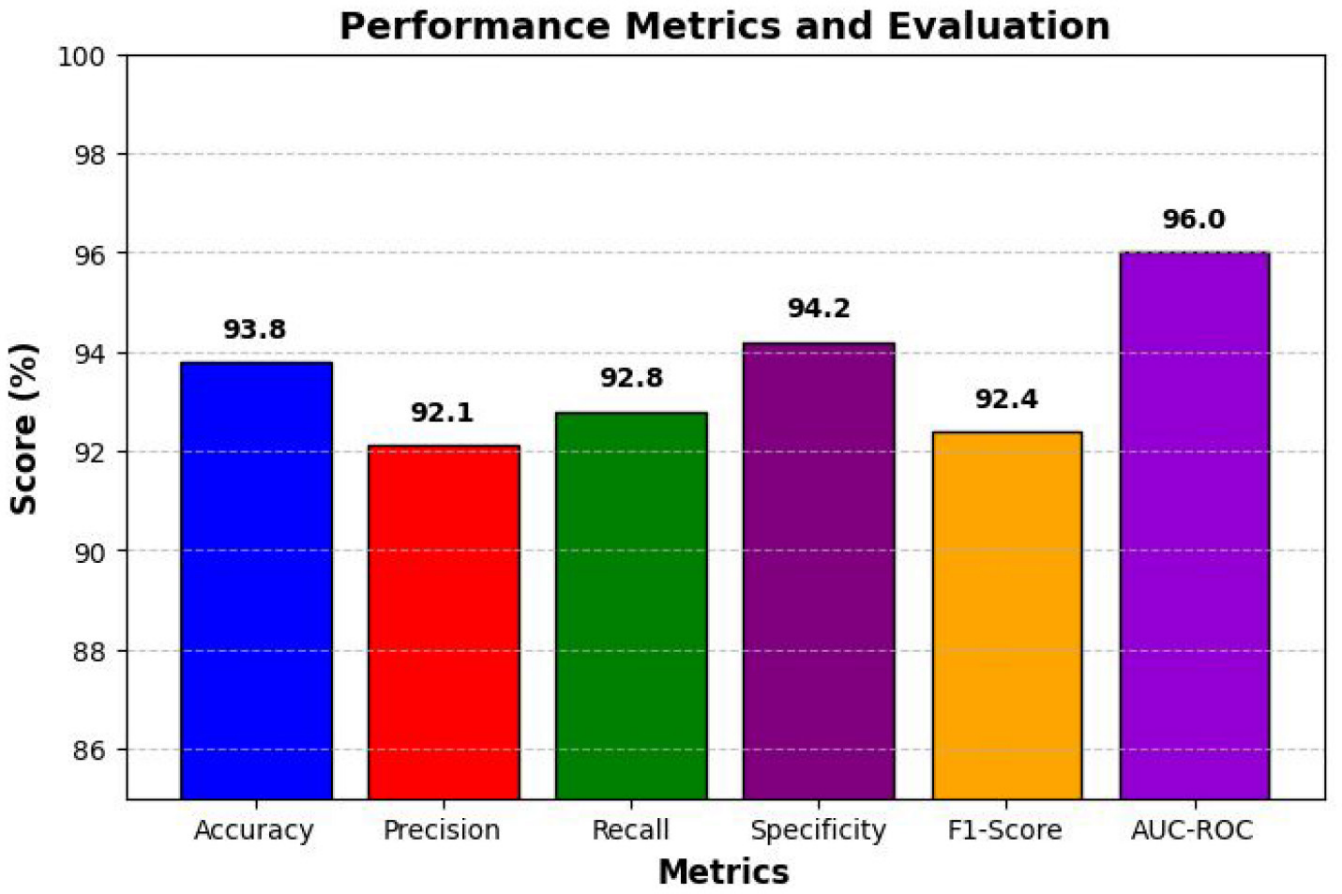
Performance analysis of the proposed model using different metrics on Messidor-2 dataset.

Accuracy computes the number of true positives divided by all cases labeled DR, which decreases the number of false-positives as illustrated in Figure 6.

Table 4 presents the performance metrics of the proposed model, achieving 93.8% accuracy, ensuring reliable classification. The recall of 92.8 indicates strong detection of DR cases, while the specificity of 94.2% minimizes false positives. The AUC-ROC of 0.96 highlights its excellent discriminatory power, confirming the model's effectiveness in automated DR detection.

**Table 4:** Performance analysis of the proposed model for Messidor-2 using different metrics.

**Metric**	**Value (%)**
Accuracy	93.8
Precision	92.1
Recall	92.8
Specificity	94.2
F1-Score	92.4
AUC-ROC	0.96

#### 4.3.1 Output visualizations and explainability using grad-CAM heatmap

**Figure 7:**
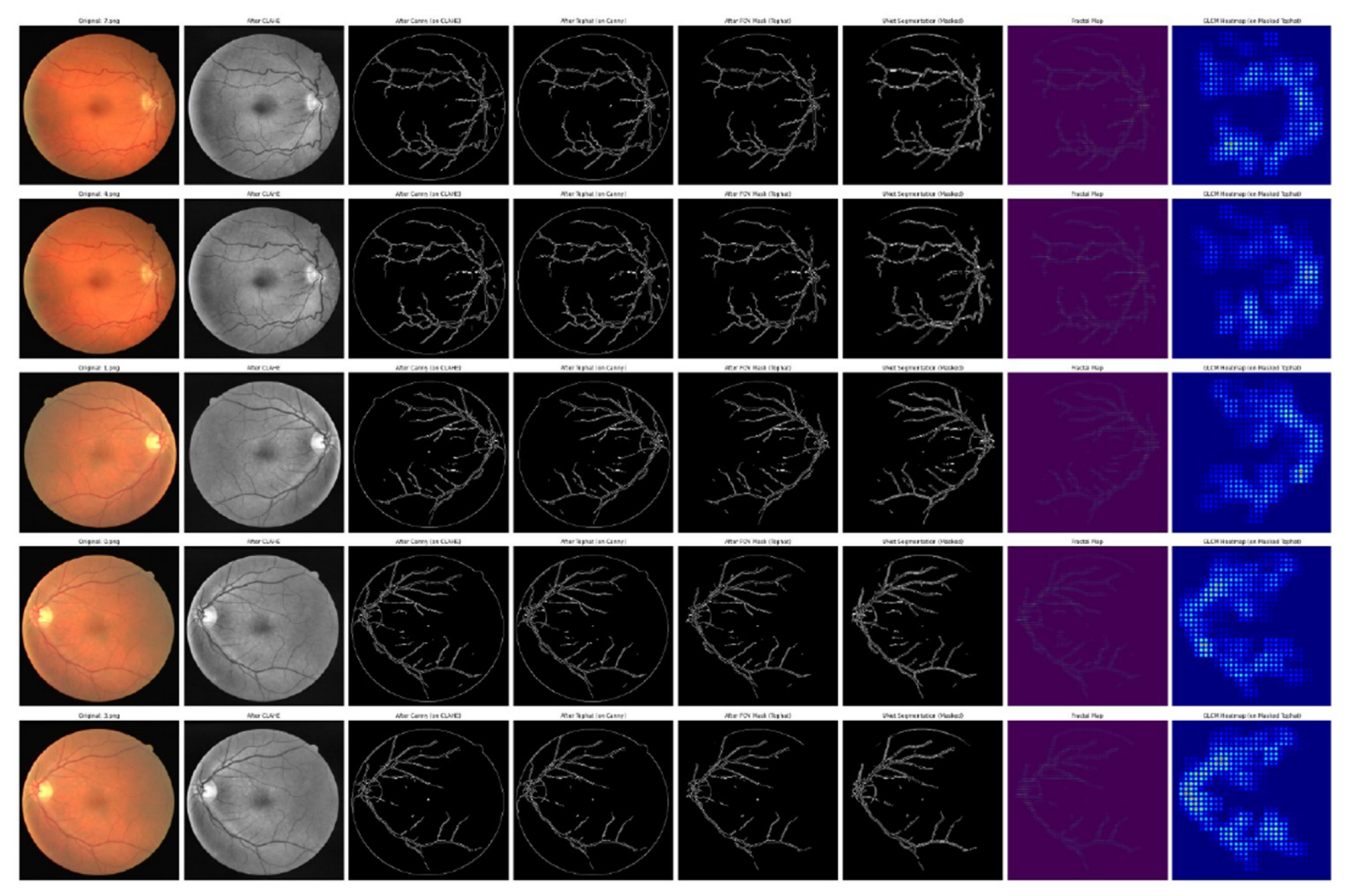
Visualization of outputs obtained during preprocessing, FDA, and GLCM.

As depicted in Figure 7, the figure depicts the visualization of outputs after preprocessing, after applying FDA and GLCM.

Gradient-weighted class activation mapping (Grad-CAM) was used to visualize the key retinal regions impacting the predictions to improve the interpretability of the suggested deep learning model. Grad-CAM generates class-discriminative heat maps that highlight the geographical regions that have the most effects on model confidence, providing insights into the convolutional layers' decision-making process.

The model mainly targets areas of high vascular complexity and optic disk boundaries. These highlighted regions are clinically relevant, as microvascular irregularities in these regions are strongly correlated with DR. The use of Grad-CAM ensures that the model's predictions align with clinically interpretable biomarkers, thereby enhancing trustworthiness for potential integration into real-world diagnostic systems.

**Figure 8:**
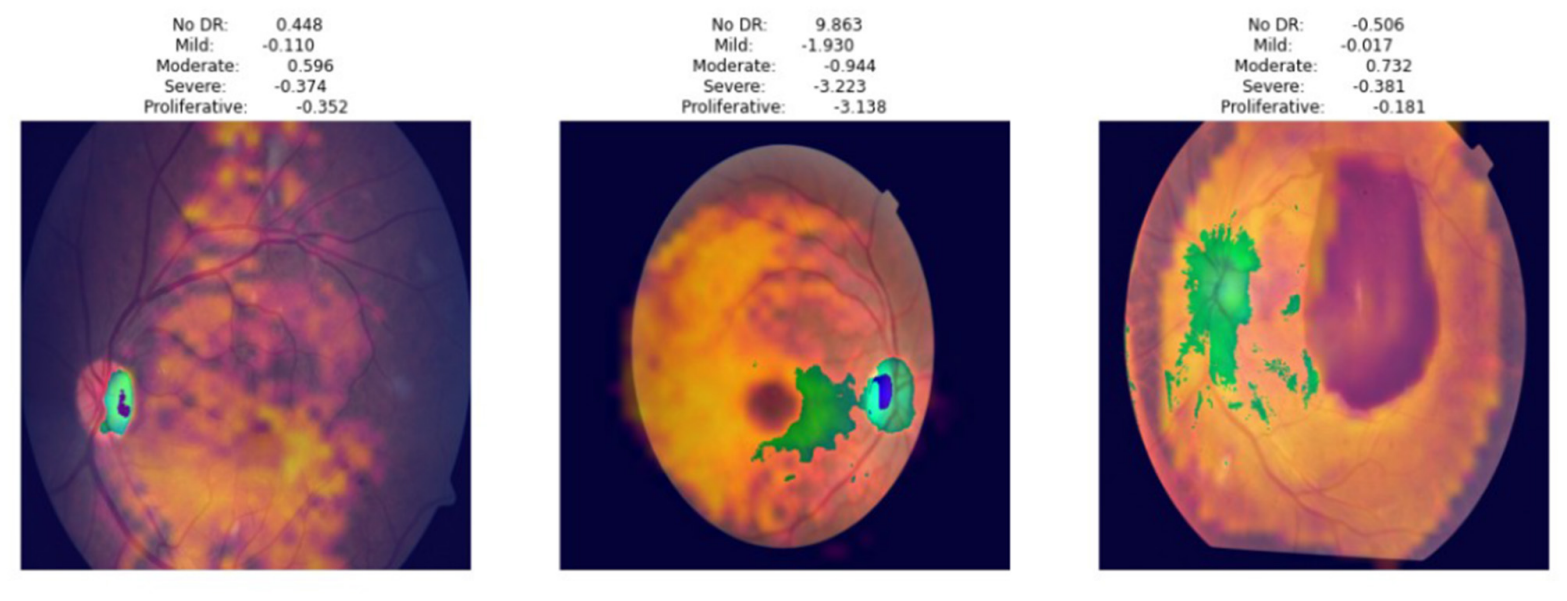
Grad-cam heatmaps obtained using the proposed model.

DR is an eye disease, and therefore, models are typically interpreted through heat maps. The Grad-CAM heat map provides a pixel-level visualization of the regions that influence the decision of the model. The green, orange, and yellow regions indicate the areas of close attention. The yellow color indicates the areas that strongly contribute to the DR class. These regions coincide with hemorrhages, microaneurysms, exudates, and areas of vascular leakage. In addition, purple/blue represents areas (outer retinal periphery in which fewer lesions are visible) with less influence on the prediction. The color distribution in Figure 8 indicates that the proposed model always attends to the regions rich in lesions, which makes predictions driven by clinically important retinal features. The green zones indicate the boundaries of the vessel, the perivascular regions, and the first lesions.

### 4.4 Comparative analysis with baseline models

**Figure 9:**
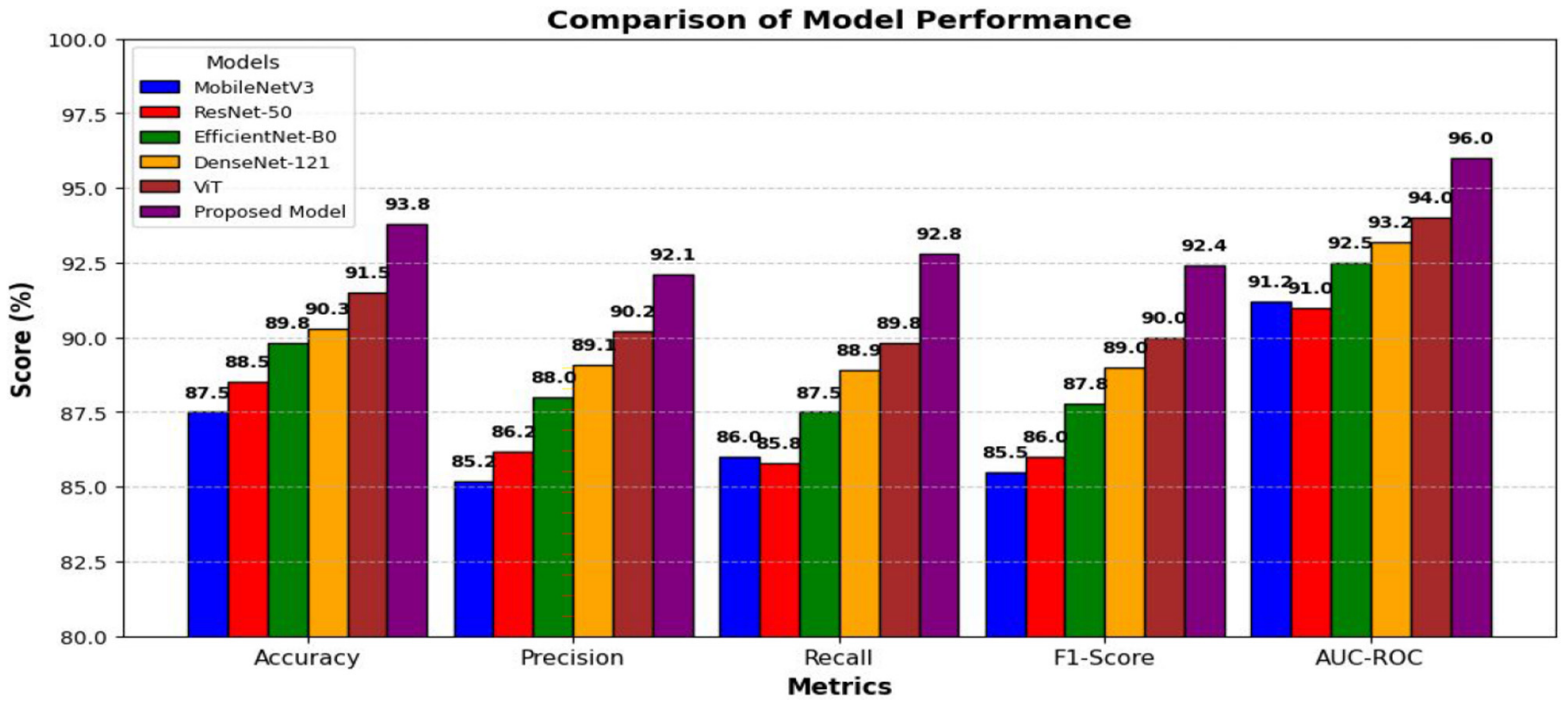
Comparative analysis with different existing models for Messidor-2.

**Figure 10:**
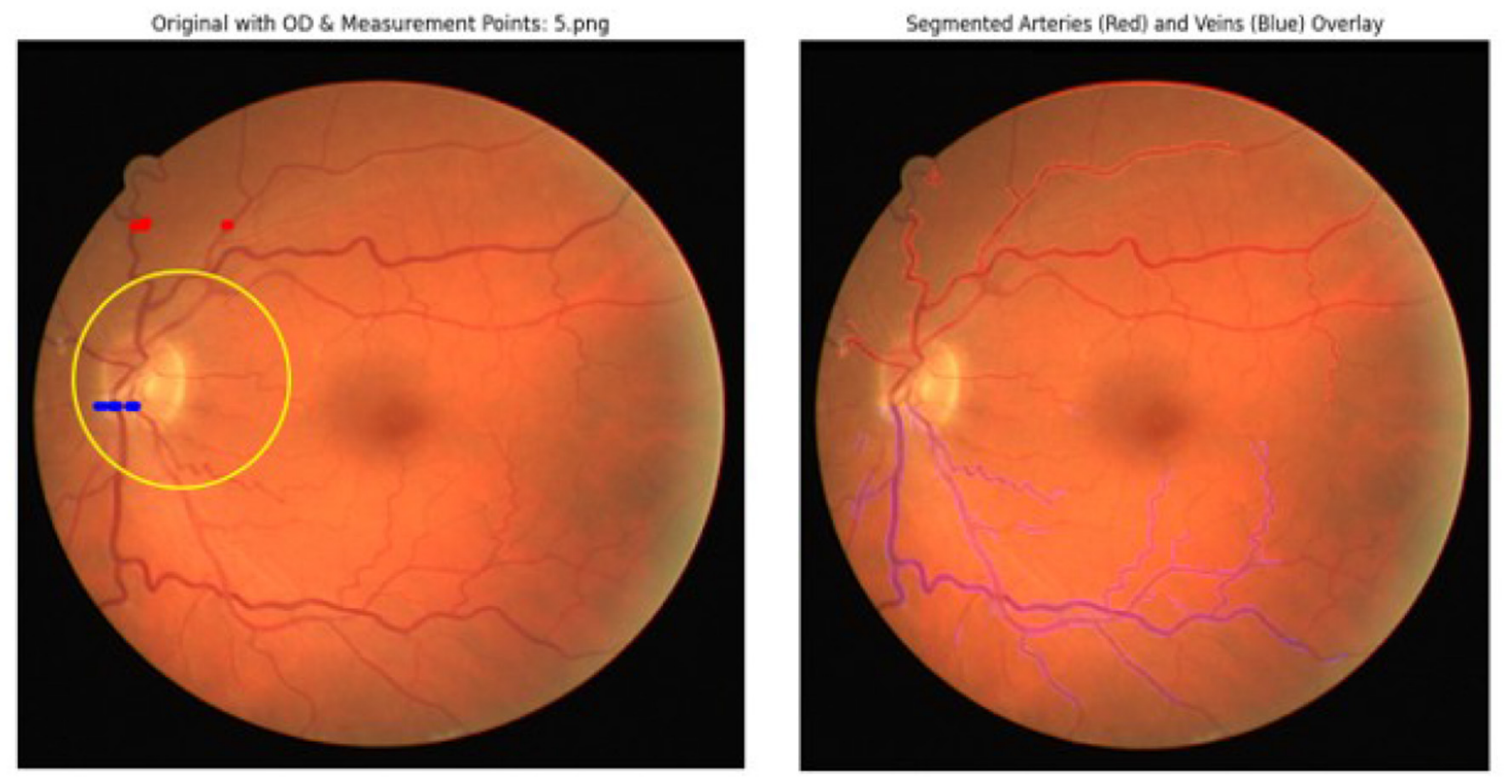
Arteries and veins for AVR biomarker.

For comparison purposes, the performance of the proposed model was also compared to the existing models in the literature, including MobileNetV3 without SE augmentation, ResNet50, EfficientNet-B0, DenseNet-121, and Vision Transformer. The suggested model surpassed all the rest, with 93.8% accuracy and 0.96 AUC-ROC as illustrated in Figure 9, proving that SE is effective in recalibrating features and dilated convolutions help increase the detection of vessel pathology. Table 5 depicts that the extraction of the vessel segmentation masks by the proposed approach is good when applying it on the retina blood vessel dataset and analyzing the performance before applying it on Messidor-2. The AVR annulus map with arteries and veins identified is given in Figure 10.

**Table 5:** Performance analysis of vessel segmentation masks extraction by CLAHE + Canny + Top-hat + U-Net on retina blood vessel dataset.

**Metric**	**Value**
Accuracy (%)	95.85
Sensitivity (%)	92.12
Specificity (%)	90.92
Precision (%)	91.45
F1-Score (%)	93.27
Dice Coefficient	0.873
IoU	0.872
AUC	0.937

### 4.5 Ablation study

Ablation experiments play an important role in estimating the contribution of different elements in deep learning models. In this work, the impact of the SE-Block attention mechanism, dilated convolutions, and preprocessing techniques on the accuracy of the proposed MobileNetV3-based retinal image classifier for DR detection is thoroughly analyzed. This analysis is achieved by the stepwise addition or elimination of significant components through an ablation study.

- **Baseline Model (MobileNetV3 Only)** There is no fine-grained vessel detection, and as a result has moderate accuracy.
- **+** **SE-Block Attention** It has improved feature representation and thereby enhances sensitivity.
- **+** **SE-Block** **+** **Dilated Convolutions** Expands the receptive field, and there is better detection of fine detailed patterns.
- **+** **SE-Block** **+** **Dilated Convolutions** **+** **CBAM** Improves the vessel visibility for mild abnormalities.
- **GLCM** Helps in capturing statistical and structural details.
- **FDA** Helps in measuring the irregularity in retinal structures.
- **AVR** The decreased AVR value helps in flagging the high-risk patients by identifying the DR severity.
- **Proposed full model** Combines all enhancements, achieving the highest accuracy, proving that each added feature significantly contributes to overall performance.

Table 6 shows the results of the ablation experiment. Baseline MobileNetV3-alone model achieves 86.5% accuracy, but has low sensitivity to fine vascular pathology. The SE-Block attention mechanism improves the accuracy to 88.3%. Dilated convolutions boost the accuracy to 88.8% by capturing the fine retinal details. Preprocessing techniques such as CLAHE and edge detection significantly enhance vessel visibility, particularly in mild DR cases, boosting contrast and structural definition. Stepwise performance improvement is evidence of the necessity for combining spatial attention, multiscale feature extraction, and advanced preprocessing techniques to reach peak classification accuracy. AVR boosts the accuracy to 93%. The entire model achieves 93.8% accuracy, indicating the overall effect of feature extraction and classification improvement. These results verify the necessity of a hybrid domain-specific and deep learning technique for medical image analysis. Also, cross-domain experiments, as in Table 7, are conducted to analyze the effect of domain shift due to variations in illumination, resolutions, and grading. Therefore, the results show the competitive performance across datasets and its ability to perform well in real-world environments. The present work focuses on publicly available datasets, but the methodology can be extended to suit naturally to hospital-based environments, which is a crucial direction for future validation.

**Table 6:** Ablation study of the proposed model using Messidor-2 dataset.

**Configuration**	**Acc**.	**Sens**.	**Spec**.	**AUC**	**F1**
MobileNetV3 baseline model	86.5	81.0	91.5	0.910	81.3
+ SE	88.3	83.8	91.1	0.926	82.1
+ CBAM	88.7	84.1	91.4	0.919	82.5
+ Dilated Convolution	88.8	86.3	91.5	0.920	82.6
+ SE + CBAM	91.2	90.6	93.0	0.935	87.1
+ SE + Dilated Convolution	91.0	90.8	91.8	0.933	87.9
+ CBAM + Dilated Convolution	91.4	91.0	92.1	0.937	88.4
+ SE + CBAM + Dilated Convolution	91.8	91.1	92.5	0.941	88.9
(above) + CLAHE	91.2	91.1	93.9	0.935	89.6
+ Canny	91.5	91.4	92.3	0.920	90.2
+ U-Net	91.7	91.5	91.6	0.923	90.6
+ GLCM	92.0	92.1	92.1	0.929	90.6
+ FDA	92.1	92.3	92.6	0.924	91.1
+ AVR	93.0	92.9	92.8	0.937	92.5
Full proposed model	**93.8**	**94.2**	**94.2**	**0.960**	**92.4**

**Table 7:** Cross-domain evaluation of the proposed model.

**Training dataset**	**Testing Dataset**	**Accuracy (%)**	**Precision (%)**	**AUC**
Messidor-2	EyePACS	98.2	97.8	0.98
APTOS 2019	Messidor-2	93.5	92.0	0.95
EyePACS	Messidor-2	93.5	92.1	0.95
Messidor-2	APTOS 2019	98.0	97.5	0.98

The results in Table 7 indicate the generalization capability across datasets that have different imaging characteristics. The results of Messidor-2 show some variation because of domain shift, and the model has high accuracy and AUC even after the transfer to APTOS 2019 and EyePACS, featuring robustness. These results indicate the suitability for deployment even in heterogeneous environments.

A five-fold cross-validation is performed on all three datasets. Table 8 shows the results and their 95% confidence intervals, indicating stability across folds. A paired t-test conducted on the 5 folds resulted in a statistically significant performance improvement when compared to the best baseline vision transformer, as *p* < 0.05. The proposed model also achieves greater than 98% accuracy on APTOS and EyePACS, with the best performance of 93.8% on Messidor-2, indicating robustness. Also, the baseline models were trained with the same preprocessing pipeline, data splits, and configuration, and the experiments are done and reported in Table 9. It indicates that the proposed work performs better than the standard CNN and transformer-based architectures for all datasets. These results also indicate the advantages of combining deep representations with vascular morphology and other descriptors.

**Table 8:** Five-fold cross-validation performance of the proposed model.

**Dataset**	**Accuracy (%)**	**Precision (%)**	**Recall (%)**	**AUC**
Messidor-2	93.8 ± 0.7	92.1 ± 0.5	92.8 ± 0.6	0.960 ± 0.008
APTOS 2019	99.2 ± 0.5	98.8 ± 0.4	99.0 ± 0.4	0.990 ± 0.004
EyePACS	98.2 ± 0.4	97.3 ± 0.3	97.5 ± 0.4	0.982 ± 0.005

**Table 9:** Comparison with baseline models under identical conditions.

**Model**	**Messidor-2 Acc (%)**	**APTOS Acc (%)**	**EyePACS Acc (%)**
EfficientNet-B0	92.3	97.2	97.1
ResNet50	92.3	96.54	96.0
ViT-Base	92.7	97.3	97.1
Proposed model	93.8	99.2	98.2

### 4.6 State-of-the-art

In medical image analysis, deep learning has been a significant advancement, especially in retinal imaging, where it allows for automated evaluation of ocular disease like DR. Because of the Messidor dataset's high-resolution and detailed retinal images, this work has investigated the utilization of retinal fundus photos.

As summarized in Table 10, the proposed approach is compared to the state-of-the-art models using Messidor-2, EyePACS, and APTOS-2019 datasets for DR detection. Performance metrics, such as accuracy, sensitivity, specificity, and AUC, are used for comparison. The proposed method achieves good performance for all the datasets. The existing methods, such as ConvNeXt, EfficientNet, and vision transformer variants, are used for the comparison. The proposed approach achieves the best performance when compared to other existing works. There will be challenges due to poor illumination, demographic bias, and the presence of artifacts. In the proposed work, CLAHE eliminates poor illumination by improving the local contrast. Canny + Top-hat suppresses artifacts and highlights the vessel and lesions. GLCM and FDA quantify vascular complexity and are robust to noise. MobileNetV3 also learns discriminative features, eliminating demographic/device bias while enhancing generalization. AVR helps in normalizing vessel caliber, eliminating the demographic bias due to age, sex, and ethnicity. SE and CBAM adaptively re-weight spatial regions, eliminating the artifacts and only focusing on lesions. Dilated convolutions magnify the receptive field, maintaining good resolution, thus helping MobileNetV3 to capture information under poor illumination and varying image quality.

**Table 10:** Comparison with the state-of-the-art models for DR detection.

**Dataset**	**Model used**	**Acc. (%)**	**Sens. (%)**	**Spec. (%)**	**AUC**	**References**
Messidor-2	**Proposed**	93.8	94.2	94.2	0.96	This work
	DR-ConvNeXt	83.6	74.0	94.6	—	Song and Wu, 2025
	DRStageNet	—	—	—	0.96	Men et al., 2023
	Swin Transformer var.	—	—	—	0.95	Yao et al., 2022; Saadna et al., 2025
EyePACS	**Proposed**	98.2	98.1	98.2	0.98	This work
	EfficientNet	—	—	—	0.90	Chetoui and Akhloufi, 2020; Yi et al., 2021
	ViT / Swin	—	—	—	0.98	Huang et al., 2024; Yang et al., 2024
APTOS 2019	**Proposed**	99.2	99.1	99.3	0.99	This work
	GPMKLE-Net	—	—	—	0.98	Zhou et al., 2023
	ConvNeXt	—	—	—	0.90	Song and Wu, 2025; Nadeem et al., 2022

### 4.7 Limitations

There is a relatively high computational cost involved both during training and inference. This will slightly affect the deployment on low-resource systems or edge devices without hardware acceleration. Also, scalability requires more optimization strategies such as model compression. The proposed work also needs further evaluation on multi-center and handheld screening devices to verify its deployment to real-world scenarios. The experiments on hospital-based data will also be done as future work, as it requires some more steps regarding domain adaptation.

## 5 Conclusion

This study suggests a novel and effective deep learning framework for DR prediction from retinal fundus images. The proposed architecture gathers local features from retinal fundus images using MobileNetV3, incorporating SE attention blocks and dilated convolutions to better capture fine-grained vascular features indicative of ocular disease such as DR prediction. Through comprehensive experiments and ablation studies, it is demonstrated that the inclusion of preprocessing techniques such as CLAHE-based contrast enhancement, Canny edge detection, and Top-hat transformation and segmentation using U-Net improves the performance. Also, the regional features captured using GLCM, the global biomarker features captured using AVR, and the FDA contribute significantly to improving model sensitivity, specificity, and overall robustness. The features are embedded in a graph-based representation using GNN that preserves vascular topology. The transformer-based cross-modal fusion integrates the multi-modal features so effectively. The model achieved an AUC-ROC of 0.96 on the Messidor dataset—outperforming conventional risk scoring systems and previously published deep learning benchmarks. Moreover, the model ensures feasibility for real-time screening in both hospital and remote settings. The AVR biomarker individually helps in DR detection after being fused with MobileNet, GLCM, and FDA features.

In future, it is aimed to expand the model's utility through multi-modal learning by integrating retinal image data with electronic health records, demographic information, and lifestyle factors to improve DR detection. Additionally, prospective validation in real-world clinical environments will be explored in collaboration with healthcare institutions to assess its diagnostic impact, usability, and integration into clinical workflows.

## Data Availability

Publicly available datasets were analyzed in this study. The datasets are available at the following links: https://www.kaggle.com/datasets/mariaherrerot/messidor2preprocess, https://www.kaggle.com/datasets/mariaherrerot/aptos2019, and https://www.kaggle.com/competitions/diabetic-retinopathy-detection.
